# Age-adjusted Charlson comorbidity index is associated with the risk of osteoporosis in older fall-prone men: a retrospective cohort study

**DOI:** 10.1186/s12877-024-05015-z

**Published:** 2024-05-10

**Authors:** Zi-Mo Pan, Jing Zeng, Ting Li, Fan Hu, Xiao-Yan Cai, Xin-Jiang Wang, Guan-Zhong Liu, Xing-He Hu, Xue Yang, Yan-Hui Lu, Min-Yan Liu, Yan-Ping Gong, Miao Liu, Nan Li, Chun-Lin Li

**Affiliations:** 1 Department of Endocrinology, The Second Medical Center & National Clinical Research Center for Geriatric Disease, Chinese PLA General Hospital, 28 Fuxing Road, Beijing, 100853 China; 2 Graduate School of Chinese PLA General Hospital, 28 Fuxing Road, Beijing, 100853 China; 3 Department of Nephrology, The Second Medical Center & National Clinical Research Center for Geriatric Disease, Chinese PLA General Hospital, 28 Fuxing Road, Beijing, 100853 China; 4 Department of Radiology, The Second Medical Center & National Clinical Research Center for Geriatric Disease, Chinese PLA General Hospital, 28 Fuxing Road, Beijing, 100853 China; 5 Outpatient Department, The Second Medical Center & National Clinical Research Center for Geriatric Disease, Chinese PLA General Hospital, 28 Fuxing Road, Beijing, 100853 China; 6 Department of anti-NBC medicine, Graduate School of Chinese PLA General Hospital, 28 Fuxing Road, Beijing, 100853 China

**Keywords:** Osteoporosis, Multimorbidity, Age-adjusted Charlson comorbidity index, Falls, Older men

## Abstract

**Background:**

There is growing evidence linking the age-adjusted Charlson comorbidity index (aCCI), an assessment tool for multimorbidity, to fragility fracture and fracture-related postoperative complications. However, the role of multimorbidity in osteoporosis has not yet been thoroughly evaluated. We aimed to investigate the association between aCCI and the risk of osteoporosis in older adults at moderate to high risk of falling.

**Methods:**

A total of 947 men were included from January 2015 to August 2022 in a hospital in Beijing, China. The aCCI was calculated by counting age and each comorbidity according to their weighted scores, and the participants were stratified into two groups by aCCI: low (aCCI < 5), and high (aCCI ≥5). The Kaplan Meier method was used to assess the cumulative incidence of osteoporosis by different levels of aCCI. The Cox proportional hazards regression model was used to estimate the association of aCCI with the risk of osteoporosis. Receiver operating characteristic (ROC) curve was adapted to assess the performance for aCCI in osteoporosis screening.

**Results:**

At baseline, the mean age of all patients was 75.7 years, the mean BMI was 24.8 kg/m^2^, and 531 (56.1%) patients had high aCCI while 416 (43.9%) were having low aCCI. During a median follow-up of 6.6 years, 296 participants developed osteoporosis. Kaplan–Meier survival curves showed that participants with high aCCI had significantly higher cumulative incidence of osteoporosis compared with those had low aCCI (log-rank test: *P *< 0.001). When aCCI was examined as a continuous variable, the multivariable-adjusted model showed that the osteoporosis risk increased by 12.1% (HR = 1.121, 95% CI 1.041–1.206, *P* = 0.002) as aCCI increased by one unit. When aCCI was changed to a categorical variable, the multivariable-adjusted hazard ratios associated with different levels of aCCI [low (reference group) and high] were 1.00 and 1.557 (95% CI 1.223–1.983) for osteoporosis (*P* <  0.001), respectively. The aCCI (cutoff ≥5) revealed an area under ROC curve (AUC) of 0.566 (95%CI 0.527–0.605, *P* = 0.001) in identifying osteoporosis in older fall-prone men, with sensitivity of 64.9% and specificity of 47.9%.

**Conclusions:**

The current study indicated an association of higher aCCI with an increased risk of osteoporosis among older fall-prone men, supporting the possibility of aCCI as a marker of long-term skeletal-related adverse clinical outcomes.

**Supplementary Information:**

The online version contains supplementary material available at 10.1186/s12877-024-05015-z.

## Background

Osteoporosis is a systemic bone disease characterized by low bone mass, deterioration of bone tissue, and disruption of bone microarchitecture, which results in bone fragility and increased fracture risk [1]. Traditionally a disease of women, osteoporosis is underestimated, underdiagnosed and undertreated in men. The lower prevalence of osteoporosis in men, when compared to women, can be explained by their higher peak bone mineral density (BMD), greater bone size, more bone trabecular, and the action of androgens [2–4]. However, with the increase in life expectancy, osteoporosis has become more prevalent in men and its poor outcome, namely fragility fracture, underscores a heavy health burden in this population. In 2017, 34% of the 2.7 million new fragility fractures occurred in Europe were in men [5]. It has also been reported that about half of the hip fractures occur before the age of 80 in men, underlying the necessity for early diagnosis and intervention [6]. In addition to osteoporosis, numerous studies have shown that falling is also a strong single risk factor for fracture in fact [7]. Approximately one-third of people all over the world aged of 65 years or older fall each year, and some may have several falls each year [8]. Thus, the primary task to reduce the incidence of fragility fractures in older men is to detect this silent disease as early as possible and to prevent falls.

Diagnosis of osteoporosis is based on BMD estimation by dual-energy X-ray absorptiometry (DXA). However, it is not widely available in many resource-constrained and underdeveloped regions. Currently, a number of assessment tools are designed to reduce the number of patients requiring a DXA screening examination and to improve access for those who most need it. Among these tools, the osteoporosis self-assessment tool for Asians (OSTA) and the Fracture Risk Assessment tool (FRAX) are the most widely used [9, 10]. However, information contained in OSTA is too small to show the heterogeneity among people of the same age and weight. And the FRAX is derived to assess fracture risk rather than to identify those with low BMD. Neither of them takes into account the characteristics of the older adults. In light of this, proposing an easy-to-use and informative tool to help assessing the risk of osteoporosis may contribute to preventing adverse events, especially for older adults at high risk of falling in hospital or community care.

Multimorbidity, commonly defined as the co-occurrence of at least two chronic conditions in the same individual, is a growing medical challenge in the aging era [11]. The global data shows that the estimated prevalence of multimorbidity in community population is 33.1%, varying in high- (37.9%), low- and middle-income countries (29.7%) [12]. The Charlson Comorbidity Index (CCI), including 17 comorbidities, was proposed in 1984 as an assessment tool for multimorbidity [13]. Since age was determined to be correlated with prognosis, the age-adjusted Charlson comorbidity index (aCCI), a modified version of CCI with the addition of age, was introduced into clinical practice in 1994 [14]. Thus far, CCI/aCCI has been extensively validated and used for survival prediction in various medical and surgical diseases, and is considered to be the gold-standard measure to assess multimorbidity in clinical research.

In recent years, evidence linking multimorbidity to skeletal-related adverse outcomes is beginning to emerge. Previous studies have shown that multimorbidity or CCI/aCCI was significantly associated with fragility fracture and fracture-related postoperative complications [15–18]. It has been reported that two thirds of osteoporosis patients had three or more comorbid diseases and the number and severity of chronic diseases accompanied will directly influence the rate of osteoporosis investigation and the treatment effect [19]. However, the role of multimorbidity in osteoporosis has not yet been thoroughly evaluated. Clarifying whether an association exists between aCCI and osteoporosis will not only address existing gaps in knowledge, but also improve the screening methods and strategies for osteoporosis, which helps prevent disease and relieve stress on the healthcare systems.

Therefore, we aimed to investigate the potential association between aCCI at baseline and the risk of osteoporosis in older men with moderate to high-fall risk in a cohort study, which would raise the possibility that indicator beyond current assessment tools might relate to osteoporosis. We hypothesized that a higher aCCI score would be associated with higher osteoporosis risk in older fall-prone patients. This understandable parsimonious algorithm may hold the great practical value for busy clinicians who obviously have the responsibility to be timely reminders for those at high risk for adverse events.

## Methods

### Study population

In this retrospective cohort study, we recruited male patients who underwent medical examinations at our hospital from January 2015 to August 2022. The number of total participants at baseline was 2124. The Morse Fall Scale (MFS) was used to measure fall risk (Supplemental Table S1), which were classified into high (≥ 45 points), moderate (from 25 to 45 points) and low (< 25 points) risk [20]. Medical records were reviewed to define osteoporosis at baseline (osteoporosis with/without fragility fracture). Participants with prescriptions of anti-osteoporotic agents (including bisphosphonates, denosumab and teriparatide; excluding calcium, vitamin D, or hormone replace therapy) were also considered to be patients with osteoporosis. In the present study, we focused on the older fall-prone men without osteoporosis at baseline, and some of them developed osteoporosis during the follow-up period. Therefore, among the original participants, those younger than 60 years old (*n* = 354), those who had previously diagnosed osteoporosis or had prescriptions including anti-osteoporosis drugs (*n* = 309), those with MFS score less than 25 points (*n* = 321), and those who were lost to follow-up or had missing data (*n* = 76) were excluded. Additionally, there are numerous causes of secondary bone loss [21]. To avoid the influence of secondary factors on our outcome, those with definite risk factors of secondary osteoporosis were excluded (*n* = 117). Finally, 947 older men with moderate to high risk of falling were enrolled in the present analysis. Informed consent was obtained from all participants. The study protocol was approval by the ethics committee of our Hospital (No. 2021–094).

### Data collection and measurements

The data of all participants was collected via standardized electronic medical records. At baseline, the data included date of birth, history of diseases, medication prescriptions, smoking status (current smoking or not), drinking status (current drinking or not) and physical activity (regular exercise or not). Trained doctors measured participants’ height and weight in light clothing. Body mass index (BMI) was calculated as weight in kilograms divided by height in meters squared (kg/m^2^). After 10 minutes of rest, systolic blood pressure (SBP) and diastolic blood pressure (DBP) were measured three times using a standard mercury sphygmomanometer, and the measurements were averaged.

After at least 8 h of fasting, the venous blood samples were collected in the next morning. The results for the following chemistry parameters were collected: glycated hemoglobin (HbA1c), total cholesterol (TC), triglycerides (TG), high-density lipoprotein cholesterol (HDL-C), low-density lipoprotein cholesterol (LDL-C), albumin (ALB), alanine aminotransferase (ALT), aspartate aminotransferase (AST), estimated glomerular filtration rate (eGFR), calcium (Ca) and phosphorus (P).

### Assessment of BMD and diagnostic criteria

Prodigy Advance DXA instrument from GE-LUNAR corporation was used to detect BMD. Then, T-scores were calculated based on BMD. According to the criteria of World Health Organization (WHO) [22], any T scores at total hip, lumbar spine, or femoral neck BMD ≤ − 2.5 were used to define osteoporosis; T scores < − 1 and > − 2.5 were used to define osteopenia. Presence of a fragility fracture was also diagnosed as osteoporosis, which was defined as low-impact fracture (resulting from a fall from a standing height or less or occurring in the absence of trauma) [23]. Besides, the 64-slice spiral CT machine from GE corporation and the 5-sample solid phantom from Mindways were also used to measure a proportion of participants’ BMD. The original images were analyzed by Mindways quantitative computed tomography (QCT) Pro software. According to the criteria of the International Society for Clinical Densitometry (ISCD) [24], trabecular volumetric BMD (vBMD) values of 120 mg/cm^3^ and 80 mg/cm^3^ are often used as thresholds to define osteopenia and osteoporosis.

### Calculation of aCCI

Based on the previous literature [13], CCI was calculated by counting each comorbidity (myocardial infarction, congestive heart failure, peripheral vascular disease, dementia, cerebrovascular disease, rheumatoid disease, peptic ulcer disease, diabetes (with/without complications), chronic pulmonary disease, liver disease (mild/moderate and severe), hemiplegia, moderate and severe renal disease, solid tumor (with/without metastasis), leukemia, lymphoma and acquired immunodeficiency syndrome (AIDS)) according to its weighted score. Then, aCCI was calculated on this basis by adding 1 point for every 10 years of age for patients over 40 years (0 point for ≤40 years, 1 point for 41–50 years, 2 points for 51–60 years, 3 points for 61–70 years, and 4 points for > 70 years) [25].

### Follow-up

The outcome was defined as a new diagnosis of osteoporosis. Participants were followed from the baseline until either an osteoporosis occurred, lost, or the cohort study was closed. Participants underwent annual physical examinations at our hospital and were followed up by face-to-face interviews. Follow-up surveys were conducted in the same way as the baseline investigation. When the on-site follow-up is not available, the self-reported disease status and medication use will be collected through telephone interviews. Incident osteoporosis was captured yearly either during the follow-up interviews by a trained physician based on the above-mentioned criteria. Once diagnosed, trained staffs would register patients with newly identified osteoporosis, and the date of diagnosis, diagnostic basis, number of fracture, fracture site, and fracture type would be recorded in detail.

## Statistical analysis

Differences between patients who developed osteoporosis and those who did not were tested using chi-square test for categorical variables. For continuous variables with normal or skewed distributions, student t test or Mann-Whitney U test was conducted. The aCCI was evaluated in the following two ways: as a continuous variable and as two categories based on the median [Low (aCCI < 5); High (aCCI ≥5)]. And differences between patients according to different aCCI levels were also tested. Cumulative incidence of osteoporosis by different levels of aCCI was assessed with the Kaplan Meier method. The Cox proportional hazards regression model was used to estimate the association of aCCI with the risk of osteoporosis. The multivariate analysis was performed for adjusting potential confounders. Hazard ratio (95% confidence interval, 95%CI) was reported in univariate analysis and multivariate analysis. Subgroup analyses were performed based on baseline age, BMI, hypertension and osteopenia. And their interactions were tested. Receiver operating characteristic (ROC) curves were constructed for aCCI and OSTA, and the area under the curve (AUC) were calculated to judge the value of aCCI and OSTA in predicting osteoporosis. Sensitivity and specificity were also calculated. A *P* value of < 0.05 (two-tailed) was considered statistically significant. Statistical analyses were performed using SPSS software version 26.0 (SPSS Inc., Chicago, IL). The drawing of figures was performed by Graphpad Prism version 9.0 and R version 4.3.1.

## Results

### Cohort characteristics

**Fig. 1:**
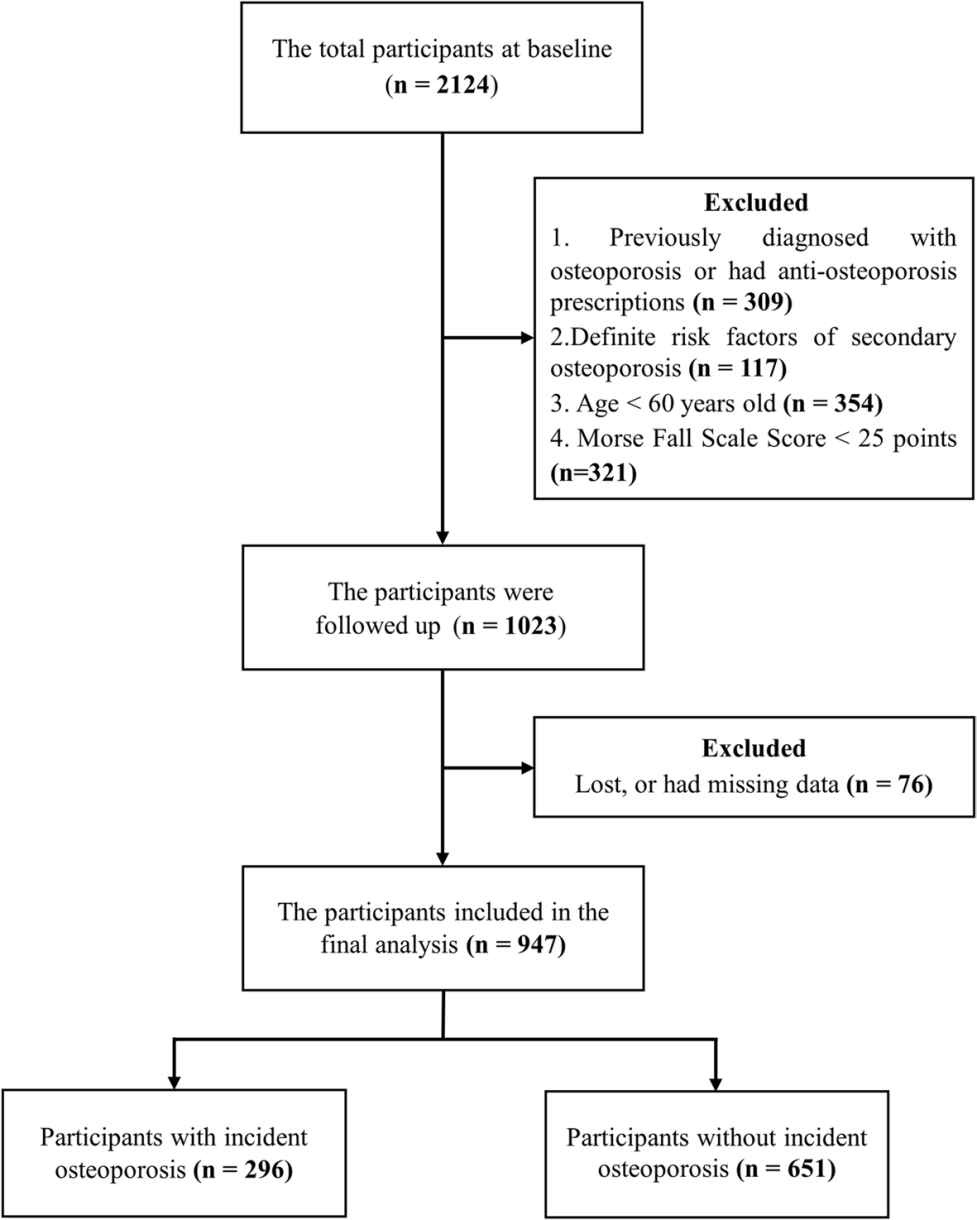
Study flow diagram

After screening, 947 older men at moderate to high risk of falling were included into the final analysis. The study flow chart was presented in Fig. 1. At baseline, the mean age was 75.7 years, the mean BMI was 24.8 kg/m^2^ and the most common comorbidity in aCCI was diabetes (39.2%), followed by peptic ulcer disease (16.8%) and cerebrovascular disease (15.7%). The prevalence of each variable in aCCI among our patients was shown in Supplemental Table S2. 531 (56.1%) patients had high aCCI (aCCI ≥5) while 416 (43.9%) were having low aCCI (aCCI < 5). During a median follow-up of 6.6 years, 296 (31.3%) participants developed osteoporosis. Among them, 34 (11.5%) were diagnosed by the occurrence of fragility fractures, and the rest were diagnosed by T scores or vBMD.

### Comparison of the baseline characteristics according to the occurrence of osteoporosis

General characteristics of the study population according to the occurrence of osteoporosis are presented in Table 1. Compared with patients who did not develop osteoporosis, patients with incident osteoporosis had higher aCCI and proportion of regular exercising, and had lower eGFR, femur neck (FN) BMD and total hip (TH) BMD. The proportion of high aCCI in patients with and without osteoporosis were 64.9 and 52.1%, respectively. There were no significant differences between the two groups in age, BMI, SBP, DBP, HbA1c, TC, TG, HDL-C, LDL-C, ALB, ALT, AST, Ca, P and the proportion of smoking and drinking.

**Table 1:** Characteristics of participants according to the occurrence of osteoporosis (*n* = 947)

	Total cohort	Non-osteoporosis	Osteoporosis	*P* value
*N*	947	651	296	–
age, years	75.7 ± 9.4	75.4 ± 9.6	76.2 ± 9.1	0.279
BMI, kg/m^2^	24.8 ± 2.9	24.9 ± 2.9	24.5 ± 2.8	0.101
Current smoker, *n* (%)	136 (14.4)	84 (12.9)	52 (17.6)	0.058
Current drinker, *n* (%)	395 (41.7)	268 (41.2)	127 (42.9)	0.615
Regular exercise, *n* (%)	660 (69.7)	436 (67.0)	224 (75.7)	0.007
SBP, mmHg	130 ± 14	130 ± 15	131 ± 15	0.347
DBP, mmHg	73 ± 10	72 ± 10	73 ± 10	0.640
HbA1c, %	6.0 ± 0.7	6.0 ± 0.7	6.0 ± 0.7	0.268
TC, mmol/L	4.09 ± 0.82	4.07 ± 0.82	4.13 ± 0.80	0.259
TG, mmol/L	1.16 (0.87, 1.59)	1.15 (0.85, 1.61)	1.17 (0.93, 1.56)	0.414
HDL-C, mmol/L	1.30 ± 0.33	1.30 ± 0.34	1.31 ± 0.32	0.681
LDL-C, mmol/L	2.56 ± 0.74	2.55 ± 0.74	2.60 ± 0.75	0.318
ALB, g/L	45.6 ± 2.7	45.6 ± 2.7	45.8 ± 2.7	0.365
ALT, U/L	17.0 (13.0, 22.0)	17.0 (13.0, 22.0)	17.0 (12.0, 21.0)	0.750
AST, U/L	20.3 ± 6.8	20.2 ± 6.2	20.6 ± 8.0	0.443
eGFR, ml/min/1.73m^2^	99.1 ± 37.4	101.8 ± 37.7	93.1 ± 36.1	0.001
Ca, mmol/L	2.31 ± 0.08	2.31 ± 0.08	2.31 ± 0.09	0.869
P, mmol/L	1.07 ± 0.14	1.07 ± 0.14	1.06 ± 0.13	0.415
FN BMD, g/cm^2^	0.898 ± 0.128	0.922 ± 0.129	0.846 ± 0.111	< 0.001
FN T-score	−0.6 ± 1.0	−0.4 ± 1.0	−1.0 ± 0.85	< 0.001
TH BMD, g/cm^2^	0.999 ± 0.138	1.025 ± 0.139	0.940 ± 0.117	< 0.001
TH T-score	0.1 ± 1.0	0.3 ± 1.0	−0.4 ± 0.9	< 0.001
aCCI	4.9 ± 1.5	4.8 ± 1.5	5.1 ± 1.4	0.004
High aCCI (aCCI ≥5)	531 (56.1)	339 (52.1)	192 (64.9)	< 0.001

*BMI* body mass index, *SBP* systolic blood pressure, *DBP* diastolic blood pressure, *HbA1c* glycated hemoglobin, *TC* total cholesterol, *TG* triglycerides, *HDL-C* high-density lipoprotein cholesterol, *LDL-C* low-density lipoprotein cholesterol, *ALB* albumin, *ALT* alanine aminotransferase, *AST* aspartate aminotransferase, *eGFR* estimated glomerular filtration rate, *Ca* calcium, *P* phosphorus, *FN* femur neck, *TH* total hip, *BMD* bone mineral density, *aCCI* age-adjusted Charlson comorbidity index

### Comparison of the baseline characteristics according to different levels of aCCI

Supplemental Table S3 depicts the characteristics of subjects by aCCI levels. Compared with patients with low aCCI, patients with high aCCI were older; had higher SBP and HbA1c; had lower proportion of smoking, drinking and regular exercising, and had lower DBP, ALB, ALT, FN BMD and TH BMD. The proportion of incident osteoporosis in patients with high and low aCCI were 36.2 and 25.0%, respectively.

### The association of aCCI with the risk of osteoporosis

**Fig. 2:**
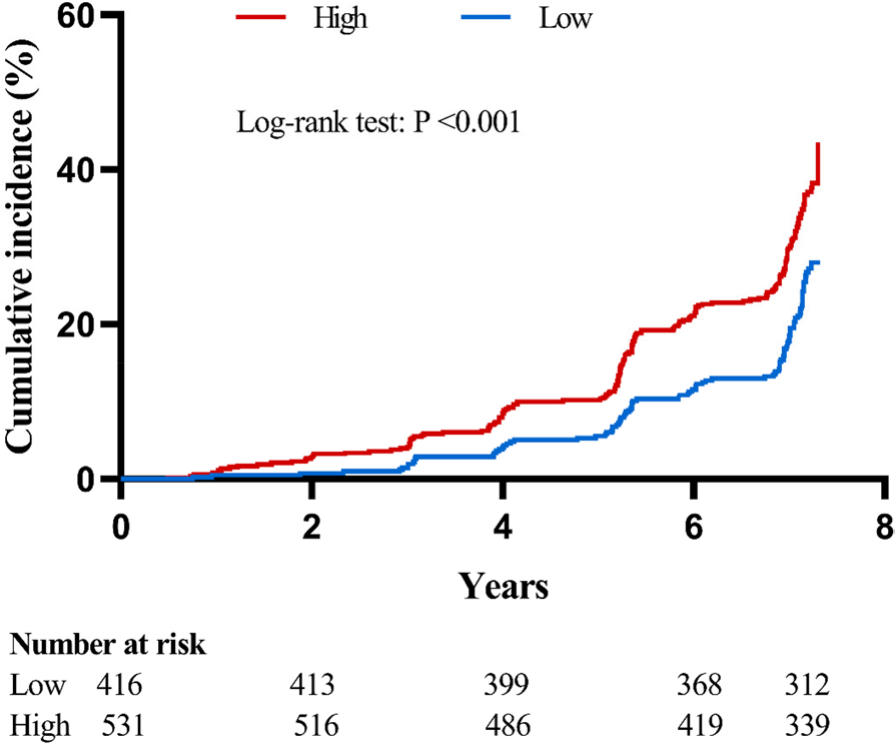
Cumulative incidence of osteoporosis by different levels of aCCI. Note: High: aCCI ≥5; Low: aCCI < 5

To identify the association between aCCI and the risk of osteoporosis, we performed univariate and multivariate analyses. Kaplan–Meier survival curves for osteoporosis according to different levels of aCCI showed that participants with high aCCI had significantly higher cumulative incidence of osteoporosis compared with those had low aCCI (log-rank test: *P* < 0.001; Fig. 2). When aCCI was examined as a continuous variable, the multivariable-adjusted model (BMI, HbA1c, TG, LDL-C, ALB, ALT, eGFR, Ca, smoking, drinking, exercising and FN BMD: model 3) showed that the osteoporosis risk increased by 12.1% (HR 1.121, 95% CI 1.041–1.206, *P* = 0.002) as aCCI increased by one unit. When aCCI was changed to a categorical variable, the multivariable-adjusted (model 3) hazard ratios (HRs) associated with different levels of aCCI [low (reference group) and high] were 1.00 and 1.557 (95% CI 1.223–1.983) for osteoporosis (*P* <  0.001), respectively (Table 2). Univariate and multivariate analyses of other potential factors for osteoporosis were shown in Supplemental Table S4.

**Table 2:** HRs and 95%CIs for osteoporosis according to different levels of aCCI

	aCCI Group		*P* value	aCCI^*^	*P* value
	Low (aCCI < 5)	High (aCCI ≥5)			
Unadjusted HRs (95% CIs)	Ref.	1.605 (1.264–2.037)	< 0.001	1.126 (1.050–1.208)	0.001
Adjusted HRs (95% CIs)					
Model 1	Ref.	1.605 (1.264–2.037)	< 0.001	1.126 (1.050–1.208)	0.001
Model 2	Ref.	1.702 (1.333–2.172)	< 0.001	1.130 (1.054–1.212)	0.001
Model 3	Ref.	1.557 (1.223–1.983)	< 0.001	1.121 (1.041–1.206)	0.002

Model 1 adjusted for BMI; Model 2 adjusted for Model 1 and SBP, HbA1c, TG, LDL-C, ALB, ALT, eGFR, Ca; Model 3 adjusted for Model 2 and smoking, drinking, exercising, FN BMD

*BMI* body mass index, *SBP* systolic blood pressure, *HbA1c* glycated hemoglobin, *TG* triglycerides, *LDL-C* low-density lipoprotein cholesterol, *ALB* albumin, *ALT* alanine aminotransferase, *eGFR* estimated glomerular filtration rate, *Ca* calcium, *FN* femur neck, *BMD* bone mineral density, *HR* hazard ratio, *CI* confidence interval, *aCCI* age-adjusted Charlson comorbidity index

^*^ aCCI as a continuous variable

### Subgroup analyses

**Fig. 3:**
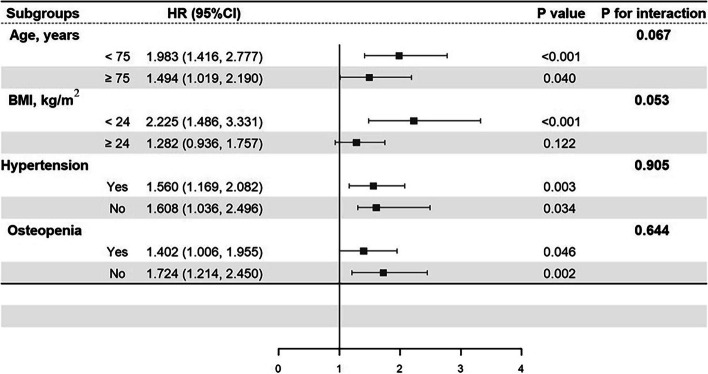
HRs for osteoporosis according to different levels of aCCI among subpopulations. Note: BMI: body mass index

Subgroup analyses were conducted to examine the association between aCCI and osteoporosis risk. When stratified by age, BMI, history of hypertension and osteopenia, all of the adjusted HRs for osteoporosis were significantly higher in the high aCCI group in all subgroups except for overweight people. There were no significant interactions of aCCI and age, BMI, history of hypertension and osteopenia on osteoporosis risk (Fig. 3).

### The performance of aCCI and OSTA in identifying osteoporosis

ROC curves were also adapted to evaluate the performance of aCCI and OSTA (− 1 and − 4 were selected as the cutoff values [9]) in screening osteoporosis in older men at moderate to high risk of falling. The area under the curve (AUC) of aCCI was 0.566 (95%CI 0.527–0.605, *P* = 0.001), with sensitivity of 64.9% and specificity of 47.9% (cutoff ≥5). The area under the curve (AUC) of OSTA was 0.535 (95%CI 0.496–0.575, *P* = 0.081), with sensitivity of 51.4% and specificity of 53.76% (cutoff < − 1); sensitivity of 17.6% and specificity of 85.6% (cutoff < − 4). A comparison of AUCs was also made between aCCI and OSTA for detecting subjects with osteoporosis. However, no significant difference was observed (*P* = 0.15).

## Discussion

This cohort study found that aCCI was significantly associated with long term risk of osteoporosis in older men at moderate to high risk of falling. Patients with high aCCI were 1.5 times more likely to develop osteoporosis as compared to patients with low aCCI. These results supported the possibility of aCCI as a marker of long-term skeletal-related adverse clinical outcomes.

In our study, we found the very high incidence of osteoporosis and fragility fracture among men aged 60 years or older. Whereas, the incidence rate reported in several current cohort studies are lower than those we reported. Petermann-Rocha et al. [26] selected 168,682 participants (51.2% men) at the age of 37–73 years with complete data for their study from UK Biobank, an ongoing prospective cohort study with over 500,000 participants recruited in 2006–2010 with multiple follow-ups. After a median follow-up of 7.4 years, 6296 (3.7%) participants were diagnosed with osteoporosis. Gourlay et al. [27] conducted a prospective cohort study of 5235 community-dwelling men aged ≥65 years without osteoporosis and hip or vertebral fracture at baseline. During 8.7 years of follow-up, 184 (3.5%) developed osteoporosis; 279 (5.3%) had a hip or vertebral fracture by the end of study follow-up (14.8 years). Several reasons may explain for the higher incidence in our study. First of all, diagnosis of osteoporosis was primarily based on DXA scan results in previous studies, while vBMD based on QCT was also used for diagnosis in our study, which greatly increased the detection rate. Secondly, the population we focused on were the older fall-prone men, who were at high risk of fracture, as fragility fracture usually occurs after a simple fall due to low-impact trauma. And lastly, worldwide variation in the incidence of osteoporosis is difficult to determine because of problems with underdiagnosis.

The CCI originally was developed to predict the risk of mortality within 1 year of hospitalization [13]. As studies move along, the predictive validity of this index with regard to mortality has been documented in numerous studies involving millions of patients with diverse diseases such as chronic heart failure (CHF) [28], arrhythmia [29], acute coronary syndrome (ACS) [30], stroke [31, 32], chronic obstructive pulmonary disease (COPD) [33], dementia [34], cancer [35–37], and COVID-19 [38], as well as patients after coronary artery bypass grafting (CABG) [39], non-cardiac surgery [40], and hip fractures (HF) [33].

However, a comorbid condition has the potential not only to impact a patient’s mortality, but also to be predisposed to the occurrence and development of another disease by a complex interaction of one or more distinct factors. This has been shown in several studies which explored whether CCI was an applicable forecasting tool for a variety of other outcomes besides mortality.

Hasan et al. [17] assessed the risk factors for postoperative complications following hip fracture surgery and found that greater number of CCI comorbidities had a higher risk of complications, providing evidence that CCI can be used preoperatively to assess the burden of comorbidity that can affect postoperative outcomes. Moreover, Clausen et al. [16] evaluated the performance of CCI in predicting major osteoporotic fracture (MOF) and HF, and reported an area under the curve (AUC) of around 0.7 in both sexes. A recent study that focused on the association between glycemic control and one-year mortality risk among patients with diabetes found that a positive association between dysglycemic measures, both hyperglycemia and hypoglycemia, and CCI [41]. In addition, CCI has also been used to predict postoperative nutritional status. Kubo et al. reported that CCI ≥2 was significantly associated with poor prognostic nutritional index (PNI) at 1 month after surgery for esophageal cancer, indicating the necessity to administer effective nutritional interventions for postoperative patients with multiple comorbidities [42].

Our results were consistent with findings from published studies, as all of them have reported that high scores were associated with mortality or considered to be a risk factor of adverse outcomes. However, different from those studies above, we chose aCCI, a modified version of CCI considering age, to measure burden of diseases rather than CCI, because aCCI has been reported to be a better predictor than CCI to some extent [43, 44]. To the best of our knowledge, the current study is the first to show the possibility of aCCI as an available marker for the risk of osteoporosis in older men who are vulnerable to fall. Notably, higher scores were identified to increase the osteoporosis risk independently after adjusting for covariates. Furthermore, the consistent association across multiple sub-populations supported the robustness of our findings.

To date, a host of researches have proved that osteoporosis, a complex biological process that involves loss of bone mass and bone strength, is closely related to age, CHF, diabetes, dementia, cancer, chronic pulmonary disease, chronic liver disease, end-stage renal disease, and many other physiological and pathological conditions [45]. Most of the diseases above are included in aCCI so that the association between aCCI and the odds of osteoporosis can be well explained. There is evidence that only 31–36% people above the age of 70 have normal bones, while the remainder suffering from osteopenia or osteoporosis [46]. With old age, bone homeostasis maintained by a balance between bone formation and bone resorption, inevitably undergoes deregulation. Oxidative stress-induced DNA damage, cellular apoptosis, and cellular senescence all get involved [46]. Long-term exposure to diabetes also changes in bone metabolism through multiple mechanisms. In particular, insulinopenia decreases bone formation by exerting an inhibitory effect on osteoblasts and their progenitor cells, whereas hyperglycemia directly affects the maturation of osteoblasts by altering gene expression [47]. In chronic kidney disease (CKD), the disturbance of the fibroblast growth factor 23 (FGF23) /1,25-dihydroxyvitamin D [1,25 (OH)_2_D]/ parathormone (PTH) axis and the irregulation of calcium and phosphate jointly lead to decreased bone mass and increased fragility fractures [48]. Besides, patients with multiple diseases are vulnerable to osteoporosis still possibly because of their limited daily activities [49] and intricate polypharmacy [50].

Given the negative impact of multimorbidity in older people, it is important to assess or manage the whole person rather than just a specific or single aspect. However, information on the impact of more than one factor is lacking prior to our study. We used aCCI, an effective, well-validated, and relatively simple tool that can easily calculate using electronic health records, to assess the overall status of the patients comprehensively. Although osteoporosis screening has a strong evidence basis, the actual screening rates are typically below 33% due to practical constraints, such as extra equipment, substantial additional cost and patient time [51]. In order to expand population screening of osteoporosis, particularly the older adults who are prone to fall——risk individuals we deem to be in greatest need of performing bone density assessment, we chose aCCI as a simple metric to signify the necessity for testing because of their probability of low BMD and high osteoporosis risk. It can be used by general practitioners and nurses in clinical practice or community care and may have broader applicability in resource-restricted and low- to middle-income countries and may prove cost-effective. We propose that for patients with a high score (aCCI ≥5), such as patients with five common comorbidities, diabetic patients older than 70 years, patients with severe liver and kidney diseases and so on, BMD should be examined as soon as possible to enable the early identification of osteoporosis, and basic treatment, such as calcium and vitamin D supplementation can be initiated as appropriate.

This is an exciting time for research with greatly enhanced capacity for assessment and development using imaging and computer technology, big data science, and machine learning. However, we have to admit that simple tools are much more valuable to busy clinicians in current clinical practice. In our study, aCCI showed a general performance in identifying osteoporosis in older fall-prone men, thus the validity of aCCI as a surrogate predictor of osteoporosis need to be explored further. In the future, more personalized assessment tools of individual patients should be developed in specific circumstances, such as younger patients without illnesses, in order to maximize the benefits of intervention while reducing waste. Finally, research that contributes to risk assessment of and prognostic judgment following complications of osteoporosis may also be a priority.

The main strengths of our study include the relatively large sample size and long follow-up time, which allowed for high statistical power and the ability to perform stratified analyses. There are also several limitations in this study. First, it was a single-center, retrospective cohort study so that a risk of selection bias could not have been avoided. Second, the possibility of omissions cannot be ruled out because the data is confirmed from the medical records. Apart from that, we were not able to control for other potential confounding factors that might have affected our results. Finally, the individuals enrolled in this study were Chinese men. Therefore, the results of our study might not be generalizable to women or population from other ethnic groups.

## Conclusions

In conclusion, we found a strong and positive relationship between aCCI and the risk of osteoporosis among older men at risk for falls. Our findings suggested that aCCI should be more broadly accepted as a clinical measure. We propose that for patients with a high score (aCCI ≥5), BMD should be examined as soon as possible to enable the early identification of osteoporosis. Further prospective studies in larger population are warranted to obtain a definitive role of aCCI in the onset and progression of osteoporosis.

### Supplementary Information

**Supplementary Material 1.**

## Data Availability

The datasets used and analyzed during the current study are available from the corresponding author on reasonable request.

## Abbreviations

1,25 (OH)_2_D: 1,25-dihydroxyvitamin D
aCCI: Age-adjusted Charlson comorbidity index
ACS: Acute coronary syndrome
AIDS: Acquired immunodeficiency syndrome
ALB: Albumin
ALT: Alanine aminotransferase
AST: Aspartate aminotransferase
AUC: Area under the curve
BMD: Bone mineral density
BMI: Body mass index
Ca: Calcium
CABG: Coronary artery bypass grafting
CCI: Charlson comorbidity index
CHF: Chronic heart failure
CI: Confidence interval
CKD: Chronic kidney disease
COPD: Chronic obstructive pulmonary disease
COVID-19: Coronavirus disease
DBP: Diastolic blood pressure
DXA: Dual-energy X-ray absorptiometry
eGFR: Estimated glomerular filtration rate
FRAX: Fracture risk assessment tool
FGF23: Fibroblast growth factor 23
FN: Femur neck
HbA1c: Glycated hemoglobin
HDL-C: High-density lipoprotein cholesterol
HF: Hip fractures
HR: Hazard ratio
ISCD: International society for clinical densitometry
LDL-C: Low-density lipoprotein cholesterol
MFS: Morse fall scale
MOF: Major osteoporotic fracture
OSTA: Osteoporosis self-assessment tool for Asians
P: Phosphorus
PNI: Prognostic nutritional index
PTH: Parathormone
QCT: Quantitative computed tomography
SBP: Systolic blood pressure
TC: Total cholesterol
TG: Triglycerides
TH: Total hip
vBMD: Volumetric BMD
WHO: World health organization
