# Supplementary material for: Age-adjusted Charlson comorbidity index is associated with the risk of osteoporosis in older fall-prone men: a retrospective cohort study
Source: BMC Geriatr. 2024 May 10;24:413. doi: 10.1186/s12877-024-05015-z (PMC11084079; doi:10.1186/s12877-024-05015-z)

**Supplemental Table S1** Number of participants per item in Morse Fall Scale

| Contents |  | Points | N (%) |
| --- | --- | --- | --- |
| History of Falling | No | 0 | 1183 (93.3) |
|  | Yes | 25 | 85 (6.7) |
| Second Diagnosis | No | 0 | 290 (22.9) |
|  | Yes | 15 | 978 (77.1) |
| Ambulatory Aid | Bed rest/Nurse assist | 0 | 1226 (96.7) |
|  | Crutches/Cane/Walker | 15 | 42 (3.3) |
|  | Furniture | 30 | 0 (0) |
| Intravenous Lock | No | 0 | 401 (31.6) |
|  | Yes | 20 | 867 (68.4) |
| Gait | Normal/Bed rest/Immobile | 0 | 503 (39.7) |
|  | Weak | 10 | 612 (48.3) |
|  | Impaired | 20 | 153 (12.0) |
| Mental Status | Oriented to Own Ability | 0 | 1231 (97.1) |
|  | Forgot Limitations | 15 | 37 (2.9) |

**Supplemental Table S2** Prevalence of each disease in Charlson comorbidity index among participants

| Variable | N (%) | | |
| --- | --- | --- | --- |
|  | Total cohort | Low（aCCI＜5） | High（aCCI ≥ 5） |
| Myocardial infarction (MI) | 45 (4.8) | 5 (1.2) | 40 (7.5) |
| Congestive heart failure (CHF) | 3 (0.3) | 0 (0.0) | 3 (0.6) |
| Peripheral vascular disease (PVD) | 25 (2.6) | 2 (0.5) | 23 (4.3) |
| Dementia | 19 (2.0) | 1 (0.2) | 18 (3.4) |
| Cerebrovascular disease (CEVD) | 149 (15.7) | 13 (3.1) | 136 (25.6) |
| Rheumatoid disease | 1 (0.1) | 0 (0.0) | 1 (0.2) |
| Peptic ulcer disease (PUD) | 159 (16.8) | 25 (6.0) | 134 (25.2) |
| Diabetes | 371 (39.2) | 97 (23.3) | 274 (51.6) |
| Chronic pulmonary disease (CPD) | 79 (8.3) | 3 (0.7) | 76 (14.3) |
| liver disease (LD) | 12 (1.3) | 1 (0.2) | 11 (2.1) |
| Moderate and severe renal disease (RD) | 42 (4.4) | 0 (0.0) | 42 (7.9) |
| Cancer (solid tumor) | 111 (11.7) | 0 (0.0) | 111 (20.9) |
| Leukemia | 1 (0.1) | 0 (0.0) | 1 (0.2) |
| Lymphoma | 1 (0.1) | 0 (0.0) | 1 (0.2) |
| Acquired immunodeficiency syndrome | 0 (0.0) | 0 (0.0) | 0 (0.0) |

**Supplemental Table S3** Characteristics of participants by different levels of aCCI

|  | Total cohort | Low  （aCCI＜5） | High  （aCCI ≥ 5） | P value |
| --- | --- | --- | --- | --- |
| N | 947 | 416 | 531 | - |
| Age, years | 75.7±9.4 | 70.9±8.8 | 79.4±8.2 | < 0.001 |
| BMI, kg/m^2^ | 24.8±2.9 | 25.0±2.7 | 24.5±3.0 | 0.012 |
| Current smoker, n (%) | 136 (14.4) | 75 (18.0) | 61 (11.5) | 0.004 |
| Current drinker, n (%) | 395 (41.7) | 210 (50.5) | 185 (34.8) | < 0.001 |
| Regular exercise, n (%) | 660 (69.7) | 316 (76.0) | 344 (64.8) | < 0.001 |
| SBP, mmHg | 130±14 | 128±14 | 131±16 | 0.002 |
| DBP, mmHg | 73±10 | 75±9 | 71±11 | < 0.001 |
| HbA1c, % | 6.0±0.7 | 5.8±0.5 | 6.1±0.9 | < 0.001 |
| TC, mmol/L | 4.09±0.82 | 4.14±0.83 | 4.04±0.80 | 0.089 |
| TG, mmol/L | 1.16 (0.87, 1.59) | 1.15 (0.86, 1.58) | 1.15 (0.86, 1.57) | 0.395 |
| HDL-C, mmol/L | 1.30±0.33 | 1.30±0.33 | 1.30±0.34 | 0.980 |
| LDL-C, mmol/L | 2.56±0.74 | 2.60±0.74 | 2.53±0.74 | 0.131 |
| ALB, g/L | 45.6±2.7 | 46.1±2.5 | 45.3±2.7 | < 0.001 |
| ALT, U/L | 19.2±10.8 | 20.2±10.8 | 18.4±15.0 | 0.042 |
| AST, U/L | 20.3±6.8 | 20.4±6.0 | 20.3±7.4 | 0.796 |
| eGFR, ml/min/1.73m^2^ | 99.1±37.4 | 98.1±29.2 | 99.8±42.7 | 0.458 |
| Ca, mmol/L | 2.31±0.08 | 2.31±0.08 | 2.32±0.08 | 0.390 |
| P, mmol/L | 1.07±0.14 | 1.06±0.14 | 1.07±0.14 | 0.110 |
| FN BMD, g/cm^2^ | 0.898±0.128 | 0.914±0.129 | 0.885±0.126 | 0.001 |
| FN T-score | -0.6±1.0 | -0.5±1.0 | -0.7±1.0 | 0.001 |
| TH BMD, g/cm^2^ | 0.999±0.138 | 1.015±0.141 | 0.986±0.136 | 0.001 |
| TH T-score | 0.1±1.0 | 0.2±1.1 | 0.0±1.0 | 0.001 |

Note: BMI: body mass index; SBP: systolic blood pressure; DBP: diastolic blood pressure; HbA1c: glycated hemoglobin; TC: total cholesterol; TG: triglycerides; HDL-C: high-density lipoprotein cholesterol; LDL-C: low-density lipoprotein cholesterol; ALB: albumin; ALT: alanine aminotransferase; AST: aspartate aminotransferase; eGFR: estimated glomerular filtration rate; Ca: calcium; P: phosphorus; FN: femur neck; TH: total hip; BMD: bone mineral density

**Supplemental Table S4** Univariate and multivariate analyses of potential factors for osteoporosis

|  | Univariate Model  HR (95%CI) | P value | Multivariate Model^*^  HR (95%CI) | P value |
| --- | --- | --- | --- | --- |
| Age, years | 1.012 (0.999-1.025) | 0.054 | 1.007 (0.993-1.020) | 0.342 |
| BMI, kg/m^2^ | 0.963 (0.925-1.002) | 0.061 | 1.018 (0.977-1.061) | 0.398 |
| Current smoker |  |  |  |  |
| No | 1.000 | - | 1.000 | - |
| Yes | 1.295 (0.960-1.747) | 0.091 | 1.464 (1.084-1.978) | 0.013 |
| Current drinker |  |  |  |  |
| No | 1.000 | - | 1.000 | - |
| Yes | 1.040 (0.826-1.309) | 0.739 | 1.103 (0.865-1.407) | 0.428 |
| Regular exercise |  |  |  |  |
| No | 1.000 | - | 1.000 | - |
| Yes | 1.363 (1.045-1.777) | 0.022 | 1.446 (1.108-1.888) | 0.007 |
| SBP, mmHg | 1.005 (0.997-1.012) | 0.196 | 1.006 (0.999-1.013) | 0.110 |
| DBP, mmHg | 1.000 (0.999-1.011) | 0.987 | 1.006 (0.995-1.018) | 0.291 |
| HbA1c, % | 0.911 (0.770-1.078) | 0.278 | 0.938 (0.791-1.112) | 0.462 |
| TC, mmol/L | 1.074 (0.934-1.234) | 0.317 | 1.056 (0.918-1.214) | 0.447 |
| TG, mmol/L | 1.033 (0.875-1.218) | 0.702 | 1.087 (0.921-1.283) | 0.322 |
| HDL-C, mmol/L | 1.068 (0.764-1.492) | 0.702 | 0.888 (0.633-1.245) | 0.490 |
| LDL-C, mmol/L | 1.085 (0.930-1.266) | 0.298 | 1.085 (0.929-1.268) | 0.303 |
| ALB, g/L | 1.003 (0.960-1.047) | 0.909 | 1.010 (0.967-1.054) | 0.666 |
| ALT, U/L | 1.001 (0.993-1.009) | 0.792 | 1.003 (0.995-1.011) | 0.488 |
| AST, U/L | 1.005 (0.989-1.020) | 0.547 | 1.009 (0.994-1.025) | 0.231 |
| eGFR, ml/min/1.73m^2^ | 0.994 (0.991-0.998) | 0.001 | 0.995 (0.991-0.998) | 0.002 |
| Ca, mmol/L | 0.791 (0.190-3.285) | 0.747 | 0.688 (0.127-3.730) | 0.664 |
| P, mmol/L | 0.654 (0.285-1.500) | 0.316 | 0.578 (0.249-1.343) | 0.203 |
| FN BMD, g/cm^2^ | 0.006 (0.002-0.019) | <0.001 | 0.006 (0.002-0.019) | <0.001 |
| FN T-score | 0.518 (0.449-0.597) | <0.001 | 0.513 (0.444-0.595) | <0.001 |
| TH BMD, g/cm^2^ | 0.009 (0.003-0.024) | <0.001 | 0.008 (0.003-0.023) | <0.001 |
| TH T-score | 0.541 (0.476-0.615) | <0.001 | 0.537 (0.470-0.612) | <0.001 |

Note: ^*^Variables adjusted for multivariate model refer to Model 3 in Table 2

BMI: body mass index; SBP: systolic blood pressure; DBP: diastolic blood pressure; HbA1c: glycated hemoglobin; TC: total cholesterol; TG: triglycerides; HDL-C: high-density lipoprotein cholesterol; LDL-C: low-density lipoprotein cholesterol; ALB: albumin; ALT: alanine aminotransferase; AST: aspartate aminotransferase; eGFR: estimated glomerular filtration rate; Ca: calcium; P: phosphorus; FN: femur neck; TH: total hip; BMD: bone mineral density

**Supplemental Figure S1** ROC curves of aCCI and OSTA in identifying osteoporosis in older fall-prone men


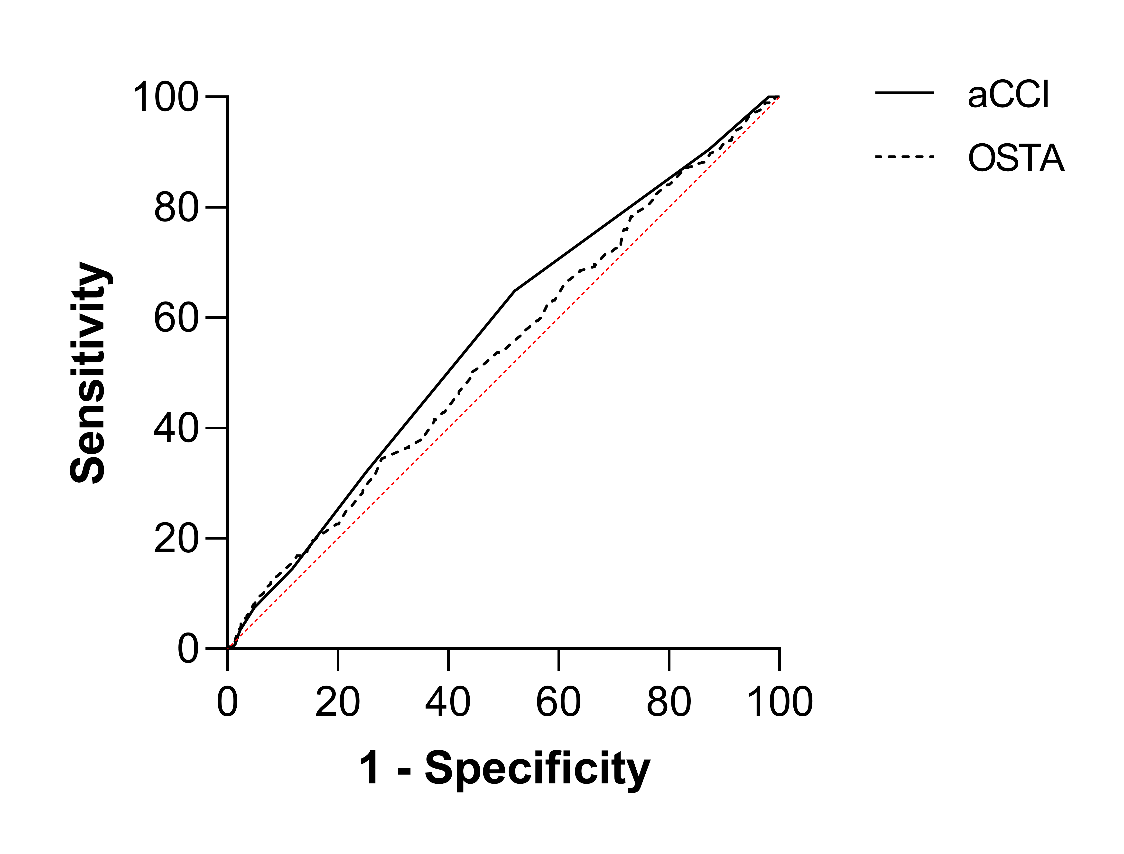

Supplement: Supplementary file 1 — Supplementary Material 1. [file 12877_2024_5015_MOESM1_ESM.docx]
